# From bodily signals to safe action: an interoceptive self-regulation loop for whole-person health

**DOI:** 10.3389/fpubh.2026.1981905

**Published:** 2026-10-15

**Authors:** Haojie Dai, Min Wang, Xiaohong Gu

**Affiliations:** 1 School of Basic Medical Sciences, Southern Medical University, Guangzhou, China; 2 School of Medicine and Nursing, Shandong Vocational University of Foreign Affairs, Weihai, China; 3 Independent Researcher, Shenzhen, China

**Keywords:** allostasis, health behavior, interoception, lifestyle medicine, patient safety, self-regulation, symptom appraisal, whole-person health

## Abstract

Health systems increasingly measure physiological and behavioral outputs, yet the transition from a bodily signal to a safe, feasible decision remains difficult to specify. We propose the Interoceptive Self-Regulation Loop (ISRL), a provisional framework linking context and exposure, bodily state, interoceptive sampling, appraisal, action, and feedback. Four measurement strata distinguish contextual inputs, functional changes, subjective signals, and clinical indicators. A safety gate operates throughout the loop through condition-specific rules, named clinical responsibility, and documented escalation and follow-up. Its purpose is to support decisions without transferring diagnosis or risk assessment to individuals. The proposed contribution is an explicit, testable account of cross-stratum discordance and evidence-responsive appraisal, rather than the invention of interoception, triage, or behavior-change support. We specify independent candidate measures of appraisal flexibility and action proportionality, illustrate the safety gate using recent-onset chest pain, and compare ISRL with a simpler combination of the common-sense model, COM-B, and identical triage guidance. Six propositions distinguish supporting predictions from the more discriminating tests of discordance, flexibility, and timely care. A bounded initial research program concerns adults in primary care, daily observations, and episode-level decisions, with prospective validation before interventional use. Repeated failure to improve prediction or decisions over the active comparator, despite adequate measurement and precision, would count against retaining ISRL as a distinct framework in that setting. Clinical safety, monitoring burden, cultural adaptation, and unequal access are explicit evaluation outcomes. ISRL remains a theory for investigation, not a validated diagnostic tool or treatment.

## 1 Introduction

Chronic disease places sustained demands on people and health services (1). Health education must help people use information within constraints of literacy, income, caregiving, and access, rather than assume that receiving a measurement makes action possible (2, 3). For decisions about seeking care, both delayed assessment and unnecessary escalation matter. A useful public-health framework should therefore specify how a signal leads to a feasible response and when self-management must give way to professional assessment. Reducing avoidable demand on emergency services is a potential outcome to test, not a demonstrated benefit of the model proposed here.

Conventional indicators remain indispensable for diagnosis and risk assessment. Their limitation arises when they are treated as the entire object of health management. A person may know a blood pressure reading but be unable to implement advice, or may report declining function without an obvious change in routine measurements. Sleep, diet, movement, psychological state, and social conditions influence the setting in which such observations arise (4–9). Monitoring outputs and supporting the processes that generate them are related but distinct tasks.

Interoception encompasses sensing, interpreting, and integrating signals from within the body (10–12). Recent whole-person-health scholarship places it at a mechanistic center and proposes a Measure, Map, Monitor, and Modulate research program (13). However, detecting a sensation does not establish its cause or determine an appropriate action. Interpretation, practical capability, material opportunity, and clinical urgency can each alter the transition from information to behavior.

We propose the Interoceptive Self-Regulation Loop (ISRL) to make these transitions explicit and testable. A safety gate, specifying clinical oversight, escalation, and follow-up, is central to its proposed implementation. Safety screening itself is established practice; the proposed addition is the joint evaluation of discordance among information sources, evidence-responsive appraisal, and action under constraints. We specify what would distinguish ISRL from established frameworks combined with the same triage guidance, and what would count against that distinction. The article develops a prospective theory, not evidence of clinical effectiveness.

## 2 Scope and approach to theory construction

The initial synthesis was a targeted narrative mapping of interoception, allostasis, illness self-regulation, behavior change, whole-person health, and self-care. Sources were selected for their ability to define constructs, explain a proposed transition, challenge a claim, establish a safety boundary, or supply a measurement approach. Conceptual papers, empirical measurement studies, evidence syntheses, and guidance from public-health or clinical organizations were eligible. Sources were not treated as evidence for an ISRL-specific causal effect merely because they supported an adjacent mechanism. Commercial testimonials, unsupported correspondences between symbolic and biological systems, and claims of universal therapeutic efficacy were excluded from the evidential argument. Disease-specific findings were retained only with their population and outcome limits.

A separately logged supplementary PubMed search was executed on 28 September 2026 using three Boolean strategies covering interoception and regulation, whole-person-health measurement, and self-management with triage or safety-netting. Searches used title/abstract terms and a publication-date window from 1 January 1900 through 28 September 2026. Supplementary Material S1 records the exact strings, returned counts, and limited retrieval scope. These are revision-stage searches, not a retrospective reconstruction of the initial search history. They support a targeted update; they do not establish exhaustive coverage. Publisher and bibliographic records, abstracts, accessible full texts, and official guidance were also used for a source-by-source citation audit, with the level of verification identified separately for each reference. A focused full-text follow-up on 29 September 2026 strengthened checks of key conceptual and measurement claims without changing the logged PubMed searches.

No systematic-review protocol was registered, screening was not performed independently in duplicate, and study-level risk of bias was not formally graded. The synthesis separates sensing, interpretation, and action; maps functional and contextual observations to these transitions; and formulates comparative tests. Supplementary Figure S1 shows how the source frameworks inform this process. Conceptual coherence and prospective incremental validity, rather than completeness of retrieval, are the appropriate criteria for judging this theory-oriented synthesis.

## 3 Conceptual foundations

### 3.1 Regulation and bodily information

Allostasis emphasizes anticipatory adjustment to demand, complementing accounts centered on regulated variables and feedback (14, 15). Sustained physiological dysregulation is associated with adverse outcomes, including mortality, but these associations do not validate any particular signal-to-action intervention (16, 17). The same measured value can arise from different combinations of illness, medication, exertion, sleep, and environment. Repeated functional observations may add context; their predictive contribution must be tested rather than assumed.

Interoception includes separable dimensions of attention, task performance, subjective sensibility, and interpretation (10–12). High confidence need not indicate accurate detection, and increased attention can be unhelpful when it intensifies threat monitoring. MAIA-2 measures eight dimensions of interoceptive awareness by self-report; it is not an objective accuracy test or a stand-alone diagnostic assessment (18). Its validation does not establish the proposed ISRL measures of appraisal flexibility or proportionality. ISRL therefore distinguishes what is noticed, what it is taken to mean, what action is feasible, and what outcome follows.

### 3.2 Appraisal and behavior

The common-sense model already describes illness representations, coping, action planning, and feedback (19). Emotion-regulation theory distinguishes strategies and their timing, while also recognizing that the usefulness of reappraisal depends on context (20, 21). COM-B explains behavior through interacting capability, opportunity, and motivation, including reflective and automatic motivational processes (22). Evidence on self-regulation components supports investigating these processes but does not establish a uniform mechanism across interventions (23). ISRL builds on these contributions and recognizes the feedback, agency, and context already represented in prior models.

Predictive-processing and active-inference accounts explain how prior expectations and sensory information can jointly shape perception and regulation (24–26). For implementation research, we propose confidence ratings as candidate behavioral indicators of belief weighting. Their relationship to computational precision would require testing; they are not established measures of a neural precision parameter. A brief task can ask a participant to rate confidence in an explanation, present credible new information, and obtain a revised rating and intended action. Repeated items can contrast relevant evidence with irrelevant reassurance. Appropriate updating is movement toward the better-supported interpretation, not simply a large change or greater reassurance. Clinical application would require independently developed items and validation.

### 3.3 Contribution relative to established frameworks

Table 1 compares ISRL with adjacent frameworks and identifies the limited contribution claimed. A particularly strong alternative is the common-sense model plus COM-B plus a condition-specific triage protocol. That combination already provides illness appraisal, practical support, and safety. Merely adding a red-flag checklist cannot distinguish ISRL from this comparator.

**Table 1:** ISRL relative to established frameworks.

Framework	Established contribution	Question for this application	Proposed ISRL test
Homeostasis and allostasis	Feedback and anticipatory regulation of bodily states	How do observations change everyday choices?	Measure transitions from signals to decisions without treating function as a specific biomarker.
Predictive processing and active inference	Inference, uncertainty, and embodied control	How can evidence updating be assessed in routine research?	Use behavioral updating tasks; do not equate confidence ratings with neural precision.
Common-sense model	Illness representations, coping, planning, and feedback	Does explicit cross-stratum mismatch add information beyond illness beliefs?	Compare prediction with and without discordance variables.
COM-B	Capability, opportunity, and motivation for action	Does separating detection and appraisal improve an already supported action plan?	Hold practical support constant and assess added decision benefit.
Whole-person health and 4M	Interoception as a mechanism; measurement and modulation agenda	Which decision transitions can be tested independently?	Specify decision, timescale, comparator, and falsification conditions.
Common-sense model plus COM-B plus triage	Appraisal, behavior support, and condition-specific safety	Is a distinct ISRL framework needed?	Test discordance and flexibility against this active comparator with identical triage rules.

The distinctive hypothesis is narrower: in a prespecified decision setting, repeated disagreement among functional, subjective, and clinical information, together with measured appraisal updating, improves prediction of appropriate action beyond the component observations themselves, illness beliefs, anxiety, capability, opportunity, prior outcomes, and the same triage rules. A component trial would additionally test whether acting on this information improves timely care without disproportionate escalation or burden. The comparator should have equivalent contact time, access assistance, and clinical safety guidance. If those additions yield no meaningful improvement, a separate ISRL framework would be unnecessary in that setting.

## 4 The interoceptive self-regulation loop

### 4.1 Six processes and four measurement strata

The loop comprises context and exposure; bodily state; interoceptive sampling; appraisal and prediction; regulation and action; and outcome and feedback. Context includes treatment, working conditions, food access, light exposure, caregiving, and social support. Bodily state includes physiological and disease processes. Sampling concerns detected internal sensations. Appraisal concerns their interpreted meaning. Action includes self-care, communication, adherence, and care-seeking. Feedback includes functioning, symptoms, clinical findings, and consequences of decisions.

Figure 1 represents reciprocal influences explicitly. Appraisal changes attention to bodily signals; action changes context; feedback changes subsequent expectations and behavior. Arrows denote candidate relationships and do not establish causal direction. The safety gate applies at every process, including the interpretation of missing or contradictory information, rather than functioning only after an action has been chosen.

**Figure 1:**
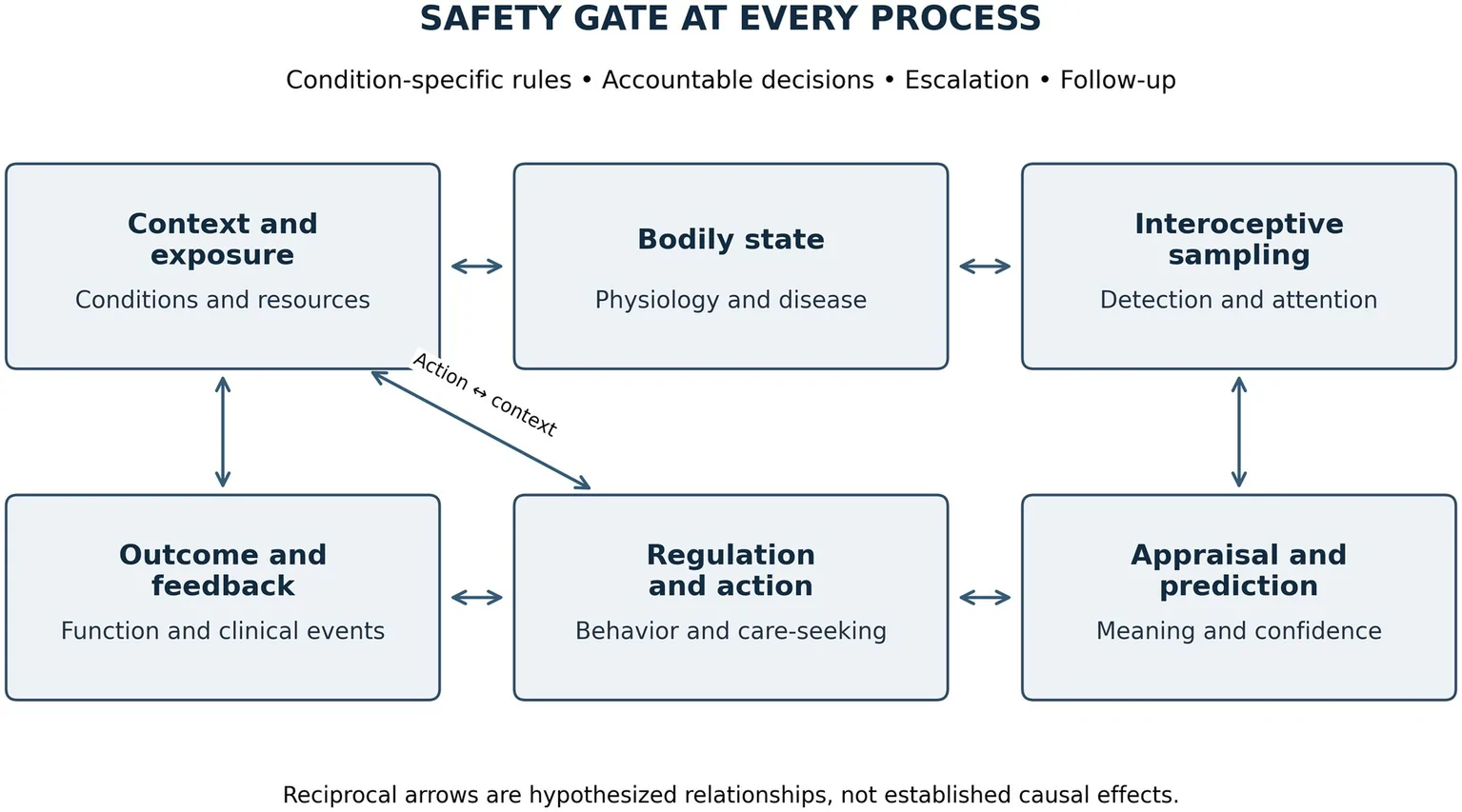
The proposed Interoceptive Self-Regulation Loop. Reciprocal influences link the six processes; action also changes context. The enclosing safety boundary applies to every process. Clinical rules and professional responsibility govern escalation and follow-up. Arrows denote hypotheses, not established causal effects.

Table 2 separates contextual inputs, functional sentinels, subjective/interoceptive signals, and objective/clinical indicators. A functional sentinel is a repeated observation of everyday functioning, not a specific early-disease biomarker. Its apparent temporal lead may reflect an already developing illness. The same event can inform different strata when the measurements differ: reported fatigue, inability to complete usual tasks, and a laboratory result should not be collapsed into one score.

**Table 2:** Measurement strata and functional-domain mapping.

Stratum	Question	Illustrative observations	Interpretive limit
1. Contextual inputs	What makes change or action possible?	Work and caregiving schedules; housing; food and service access; light exposure; treatment.	The environmental domain belongs primarily here. Its effects on sleep or participation can also appear in stratum 2.
2. Functional sentinels	What has changed in daily functioning?	Sleep regularity and daytime function (5.1); eating and bowel patterns (5.2); mobility and recovery (5.3); cognition, mood, and participation (5.4); functional effects of environmental constraints (5.5).	Repeated changes are nonspecific. Do not count an exposure itself as dysfunction or assume that a functional change precedes disease.
3. Subjective and interoceptive signals	What is sensed and how is it interpreted?	Symptom reports; confidence in an explanation; attention; MAIA-2 dimensions; evidence-updating tasks.	Distinguish attention, accuracy, appraisal, and confidence; self-report is not objective diagnostic evidence.
4. Objective and clinical indicators	What clinical information is available?	Clinically appropriate examination, vital signs, tests, diagnoses, and guideline-defined risk features.	Decision priority is condition-specific. Normal or missing measurements do not automatically negate concerning symptoms.

### 4.2 Constructs and bounded measurement

Sampling is the detection of and attention to internal signals. Appraisal is their interpreted meaning. Flexibility is appropriate updating when credible evidence changes. Capability is the skill and physical capacity to perform an action. Opportunity is its material and social feasibility. Proportionality is agreement between the urgency or intensity of action and a contemporaneous, independently adjudicated clinical reference. Feasibility and proportionality are separate: an appropriate recommendation can remain inaccessible.

For an initial measurement study, flexibility could be assessed with standardized vignettes that sequentially introduce risk-relevant information. A panel would prespecify defensible interpretations and updates before participant responses are collected. Scores would distinguish failure to update, indiscriminate switching, and appropriate stability when information is uninformative. Proportionality would be scored on separate cases or actual decision episodes against an independent panel’s urgency category, using information available at that time. Under-escalation and over-escalation would be reported separately. Panel disagreement and indeterminate cases would be retained and analyzed rather than converted into artificial certainty. Later diagnoses would be used for sensitivity analyses, not to redefine all earlier decisions with hindsight. Research adjudicators should use standardized contemporaneous clinical summaries while blinded to derived ISRL scores, allocation, actual actions, and later outcomes. Actual actions would then be compared with the independently assigned urgency category. Identical adjudication and follow-up should cover referred and non-referred episodes in both arms.

For P2, discordance would be a prespecified directional pattern, not an unweighted difference between unlike scales: for example, declining daily function paired with low reported concern or stable selected clinical observations. Component definitions, observation windows, reliability requirements, and missing-data rules would be fixed before outcome inspection. Stable selected measurements would not establish absence of disease.

Separate instruments would assess anxiety, health literacy, intolerance of uncertainty, personality, and existing illness beliefs. As illustrative development criteria, moderate associations with neighboring constructs may be compatible with distinctness; an absolute latent correlation below 0.80 would be a provisional screening criterion, not proof. Replicated correlations of 0.85 or more, failure of a separated measurement model, and no incremental prediction would together argue for merging or dropping the construct. Decisions would use confidence intervals for latent correlations and prespecified competing measurement models; values between these ranges or intervals spanning the proposed boundaries would remain inconclusive. These proposed thresholds are not validated ISRL standards; a measurement protocol must justify and preregister them, account for reliability and uncertainty, and examine invariance across languages and populations.

The framework can describe seconds-to-minutes, days-to-weeks, and longer-term processes, but a single study should select one decision and timescale. An initial testbed is adults in primary care with recurrent symptoms, after guideline-based assessment of immediate danger, using daily observations and episode-level decisions. A proposed primary prediction window is the next 24 h, with a seven-day window specified as secondary before analysis. This restriction provides a falsifiable first application; it does not validate pediatric, emergency, or long-term disease-progression applications.

## 5 Functional domains and action skills

The first four domains below supply examples of functional sentinels in stratum 2 of Table 2. The fifth identifies environmental exposures in stratum 1 and their functional expressions in stratum 2; this distinction avoids treating context itself as a symptom. These domains guide selective measurement rather than a requirement to assess every domain at every visit.

### 5.1 Sleep and circadian regularity

Sleep duration, timing, regularity, and daytime impairment are distinct observations. Consensus work supports regular timing, and prospective evidence associates greater regularity with lower mortality risk (27, 28). Such findings do not demonstrate that changing timing reduces mortality or that one bedtime is universally appropriate. Shift work, caregiving, sleep disorders, and treatment must inform interpretation. Timing variability and daytime functioning belong in the functional stratum; schedules and light exposure belong in the contextual stratum.

### 5.2 Eating and gastrointestinal function

Appetite, tolerance, meal patterns, and bowel function can describe daily change. Dietary guidance concerns overall patterns (4). The republished PREDIMED analysis supports the potential benefit of specified Mediterranean dietary patterns in a defined high-risk population, with acknowledged departures from the original randomization protocol (29). It does not validate self-directed dietary treatment for new symptoms. Proposed fasting or restrictive diets should trigger a clinician-led check of disease, medication, nutritional risk, and relevant contraindications through the safety gate. Persistent or concerning symptoms follow condition-specific assessment pathways.

### 5.3 Movement and physical function

Usual activity, mobility, recovery, and participation provide functional observations; access to safe space or assistance is contextual. Physical-activity guidance supports benefits across several health outcomes while distinguishing populations and abilities (30). A plan should specify activity, timing, amount, progression, and alternatives according to clinical suitability. Reduced activity may be a consequence of disease rather than its cause; neither motivational advice nor additional monitoring resolves that ambiguity by itself.

### 5.4 Psychological and cognitive function and social participation

Mood, distress, cognitive fatigue, difficulty concentrating, and disrupted social roles can affect interpretation and action. Cognitive fatigue or “brain fog” should be recorded as a reported or functional difficulty, with its timing and impact, rather than as an etiological diagnosis. Recent mobile-health work in complex chronic illness includes fatigue and brain-fog outcomes, illustrating a context for repeated measurement without establishing general applicability (31). New cognitive changes require clinically appropriate assessment; psychological explanations should not be presumed from normal routine measurements.

### 5.5 Environment and opportunity

Housing, employment, financial strain, discrimination, caregiving, and access to services shape feasible behavior (3). Light exposure also illustrates a contextual influence on sleep and circadian regulation (32). This fifth domain is primarily contextual, not an additional symptom score. Its functional expressions, such as disrupted sleep or participation, can be recorded in the second stratum. Table 2 makes this distinction explicit so that environmental barriers are not mislabeled as personal dysfunction.

### 5.6 Attention and appraisal skills

Attention training and contemplative practices are candidate ways to observe experience and reconsider interpretations. A systematic review found small improvements in anxiety, depression, and pain from mindfulness programs compared with nonspecific active controls, without evidence of superiority to other active treatments (33). Harms reporting was sparse, so the review cannot establish safety across populations. That evidence does not establish improved ISRL flexibility or timely care. Those mediators and outcomes require direct testing. A person may instead benefit from less monitoring, external support, or ordinary clinical treatment.

The safety gate should include a pre-practice review of vulnerability, treatment needs, and the ability to stop or obtain support. Panic, trauma-related destabilization, emerging psychotic symptoms, or deterioration during a practice warrant modification or cessation and appropriate professional assessment. These are proposed safeguards, not claims that all people with those histories must avoid every practice. Attention exercises, breathing, fasting, movement, and dietary change must not postpone indicated care. Adverse effects, distress, and disengagement should be measured alongside possible benefits.

## 6 An operational safety gate

### 6.1 Specification and accountability

A safety gate requires inputs, decision rules, outputs, and named responsibility (Table 3). A clinical governance group should select current condition-specific guidance, define the intended population, approve local referral routes and response times, and version the protocol. Patient and community representatives should help assess comprehension, feasibility, and burden. Clinical thresholds are not generated by an ISRL score or an artificial-intelligence system. Where guidance is uncertain, the protocol must identify who makes the clinical judgment and how uncertainty is documented.

**Table 3:** Minimum specification of a safety gate.

Decision	Inputs and rule	Output	Accountable role
Select the pathway	Population, condition, current guideline, contraindications, local resources; approve and version a defined pathway.	Documented scope, thresholds, response times, and escalation contacts.	Clinical governance lead, with relevant specialists and community input.
Identify an urgent assessment need	Onset, trajectory, red flags, clinical observations, medication, and missing data; apply the approved condition-specific rule.	Emergency, urgent, or other clinically justified assessment; self-management paused when indicated.	Qualified clinician or locally authorized triage professional; emergency service receives urgent handover.
Resolve discordance	Compare subjective, functional, and clinical information; do not average away a red flag or substitute a consumer display for required assessment.	Reasoned assessment and documented uncertainty, including possible alternative causes.	Responsible clinician; participant supplies observations without being assigned diagnosis.
Make the plan feasible	Ability, language, transport, cost, caregiving, and available services; check whether the recommendation can be completed.	Named referral recipient, access assistance, and follow-up if contact or attendance fails.	Care coordinator and responsible clinician; participant or invited supporter confirms feasibility.
Review benefit and harm	Recurrence, deterioration, missed care, non-indicated referral, distress, and monitoring burden; apply review and stopping rules.	Continue, reduce, stop, or revise the plan; audit and revalidate material protocol changes.	Named reviewing professional and clinical governance team.

Inputs include symptom onset and trajectory, relevant red flags, baseline conditions, medication, available clinical observations, and whether a referral can actually be completed. Children, pregnancy, frailty, disability, and multimorbidity may require different pathways; an adult example must not be generalized by simply retaining the same thresholds. Missing data are recorded explicitly. A reassuring subjective report cannot override an independently actionable clinical abnormality; a normal consumer-device reading cannot negate a concerning symptom pattern. Unresolved discordance leads to the appropriate clinical assessment pathway rather than averaging the signals into reassurance.

### 6.2 Worked example involving recent-onset chest pain

Consider an adult reporting new chest discomfort with a normal heart-rate display. The display does not establish absence of acute disease. A qualified clinician or locally authorized triage professional evaluates the history and applies the relevant pathway. In the NICE example, suspected acute coronary syndrome with current chest pain warrants emergency hospital referral. If pain has resolved but occurred within the preceding 12 h, an abnormal or unavailable resting 12-lead ECG also warrants emergency referral. For suspected cases who are currently pain-free and have no reason for emergency referral, urgent same-day assessment is indicated if pain occurred in the preceding 12 h with a normal resting 12-lead ECG, or if the last episode was 12–72 h earlier. A normal ECG alone does not exclude acute coronary syndrome, and obtaining it must not delay an indicated transfer. If acute coronary syndrome is not suspected, other potentially life-threatening causes still require consideration (34). This illustrates an existing adult guideline, not a complete chest-pain algorithm or a universal protocol.

ISRL adds documentation of the transition: what was sensed; whether a reassuring display altered the person’s interpretation; what action was recommended; what transport, language, or caregiving barrier prevented it; and whether a receiving service accepted the handover. The clinical decision remains governed by the same guideline in both ISRL and comparator care. If a barrier prevents the recommended action, the service must activate an alternative route and record the unresolved risk. If assessment excludes immediate danger, an individualized follow-up plan records who reviews recurrence or deterioration and when. Completion of a symptom diary or an appraisal exercise is never a prerequisite for urgent assessment. This acute presentation illustrates when the proposed monitoring workflow must stop; it is not an inclusion scenario for the initial recurrent-symptom cohort in Section 4.2.

### 6.3 Evaluation before an intervention trial

Initial evaluation should use independently adjudicated cases and then prospective observation without allowing an unvalidated tool to alter care. The reference concerns whether escalation was indicated using the information available at the time; eventual diagnosis is a separate outcome. Report sensitivity for indicated escalation, specificity among episodes without such an indication, positive predictive value, missed or delayed care, unnecessary referrals, anxiety, burden, and adverse events. False-positive referrals are not equivalent to referrals that fail to produce a diagnosis, because appropriate exclusion of serious disease can justify assessment.

Before recruitment, a condition-specific protocol must define an acceptable upper bound on missed indicated care and on excess non-indicated referrals, using clinical harm, service capacity, and patient priorities. No universal percentage is justified by this conceptual paper. Report confidence intervals against these bounds, stratify by clinical risk and access, and examine net benefit across plausible thresholds. A pilot advances only if its safety precision and feasibility meet the prespecified criteria. Otherwise thresholds or workflow require redevelopment and fresh validation. Service congestion, repeated testing, and distress are costs of over-escalation; optimizing sensitivity alone is insufficient.

## 7 Testable propositions and research program

### 7.1 Comparative predictions and failure conditions

Table 4 distinguishes propositions with different evidential roles. P1 and P4 are supporting predictions compatible with existing literature; their confirmation would not establish ISRL’s novelty. P5 concerns context and the limits of individual-level intervention. P2, P3, and P6 bear more weight in distinguishing the model from a simpler combination of established frameworks. Each protocol should prespecify population, decision, time window, comparator, primary metric, minimally worthwhile improvement, and acceptable harms before outcome data are examined.

**Table 4:** Propositions and comparative failure conditions.

Proposition and role	Prespecified comparison	Predicted contribution	Evidence against the proposition
P1 Temporal sensitivity Supporting	Prior outcomes and clinical severity versus those variables plus repeated functional change; next-day primary and seven-day secondary windows in the initial testbed.	Added out-of-sample prediction of prespecified functioning or decision outcomes.	Precise estimates exclude the prespecified worthwhile improvement; contemporaneous-only association weakens an antecedent interpretation.
P2 Discordance Core discriminator	Active-baseline predictors including the same raw components and comparable nonlinear/interaction modeling versus that baseline plus a prespecified directional discordance representation.	Discordance adds calibrated prediction of adjudicated decision needs or subsequent impairment.	Patterns fail external replication or add no worthwhile prediction beyond their component measurements and the active baseline.
P3 Appraisal flexibility Core discriminator	Illness beliefs, anxiety, literacy, attention, capability, and opportunity versus those variables plus an independently measured updating score.	Appropriate updating predicts proportionality on separate cases or later episodes.	Distinctness fails or precise estimates exclude added decision information; common-method correlations alone do not support the proposition.
P4 Supported action Supporting	Education alone versus education with planning, feedback, and opportunity support, with safety maintained in both groups.	Better adherence or participation where the support addresses a measured barrier.	Adequately implemented and precise comparisons exclude the minimum worthwhile benefit; a positive result does not distinguish ISRL.
P5 Opportunity moderation Boundary and equity	Prespecified variation in access and resources in the relationship between individual skill and action.	Skill translates less effectively into action under constrained opportunity; addressing barriers may reduce this gap.	Precise estimates show invariance despite reliable opportunity measurement. Equity also fails if benefit widens prespecified gaps in care or burden.
P6 Safety across transitions Core discriminator	Matched common-sense-model/COM-B/triage care, including completion tracking and follow-up, versus the same care plus structured discordance review and appraisal-updating support.	Less delayed indicated care without excess non-indicated escalation, distress, or unequal burden beyond prespecified limits.	No worthwhile improvement with adequate precision and fidelity, or unacceptable harm. Necessary triage is never withheld from the comparator.

For the proposed adult primary-care testbed, the core framework would lose its justification as a distinct approach if adequately measured, externally validated studies jointly show that discordance adds no useful prediction, flexibility adds no useful decision information, and the corresponding component intervention produces no worthwhile benefit over matched common-sense-model/COM-B/triage care. This requires estimates precise enough to exclude prespecified worthwhile effects; nonsignificant findings with wide intervals are inconclusive. Replication across independently sampled services strengthens the inference. Positive P1 or P4 findings alone cannot rescue the distinctive claim. Demonstrated harm would stop deployment even if a predictive proposition were supported. Rejection here is bounded to the tested domain, rather than an assertion about all possible forms of self-regulation. Mixed results require component-specific revision: a precise failure of P2 or P3 removes that incremental claim even if another component remains useful. P6 benefit without the predicted processes supports the service package but does not by itself validate the distinctive ISRL explanation.

### 7.2 Measurement and temporal studies

Measurement studies should first test reliability and the separation of constructs described in Section 4.2. The Whole Person Health Index is a nine-item self-report measure described in recent work (35). A 2026 psychometric study supports a dominant general factor, with mixed single-factor fit and age-related differential functioning of the self-rated-health item (36). These findings do not validate ISRL or replace its decision measures. If the index is used as an outcome, overlap between its lifestyle items and study predictors should be addressed to avoid attributing shared item content to added predictive value. A systematic review of interoception and lifestyle factors reports limited-quality evidence and the need for longitudinal research (37). A scoping review found six measures with varying domain coverage; environmental and socioeconomic domains received less attention in the self-report measures than in the conceptual models reviewed (38). Digital phenotyping remains heterogeneous; interoception itself was assessed in only a small subset of the studies in a recent review (39). These sources support restrained measurement development, not an established multidimensional ISRL score.

An 8–12-week cohort could collect a small daily set of function, context, symptoms, and actions, with additional brief records at relevant episodes. Prior function, symptom severity, treatments, and objective indicators should enter prespecified autoregressive baselines before ISRL variables are added. Analyses should separate within-person from between-person variation and use forward temporal validation and an independent service or cohort. The P2 baseline must include the same raw component measurements, with comparable nonlinear terms, interactions, and tuning opportunities. Data-derived transformations must be estimated in training data only. Any gain from derived discordance would support the utility of that representation, not independent biological information. Existing mobile-health findings show that physiological data can add short-term predictive information in a specific chronic-illness population; they do not establish ISRL’s proposed causal sequence (31).

Reverse causality remains central. Record the onset of illness, treatment changes, and care episodes; evaluate prespecified lag lengths; test reverse-lag models; and perform sensitivity analyses excluding observations close to identified acute events. A causal diagram should distinguish possible confounders from mediators so that adjustment does not automatically remove the pathway being tested. Negative-control exposures or outcomes should be used only when their assumptions are defensible. If a functional change predicts only contemporaneous deterioration, it should be described as a marker, not a causal antecedent. Predictive usefulness and causal interpretation are separate conclusions.

To reduce fatigue, use participant-selected reminder times, a short core questionnaire, optional low-frequency modules, accessible paper or telephone routes, and clearly explained pauses. Collect reasons for missed entries and withdrawal without penalizing participants. Examine whether missingness depends on symptoms, anxiety, literacy, or access; compare retained and lost participants and use justified missing-data sensitivity analyses. High completion among a selected subgroup does not establish feasibility for the target population. Monitoring frequency itself should be treated as a possible intervention and source of harm.

### 7.3 Comparative intervention testing

After measurement and safety validation, a component trial can compare common-sense-model/COM-B support plus guideline triage with the same package plus discordance review and appraisal-updating support. Both arms should have equivalent contact time, opportunity assistance, referral access, completion tracking, and follow-up of uncompleted actions. The randomized contrast concerns structured discordance review and appraisal-updating support rather than additional basic follow-up. Prespecify a functional outcome and an adjudicated decision outcome, with delayed indicated care, unnecessary escalation, anxiety, workload, adverse events, and costs as safety or secondary outcomes.

Mediation analysis should examine whether changes in the proposed processes precede decision changes, with its causal assumptions stated. Benefits without the predicted mediators may support the service intervention while weakening the ISRL explanation. A comparison against education alone may test P4 but cannot by itself establish theoretical superiority. P6 should test the additional implementation of safety across decision transitions against identical clinical rules, not compare safe care with withdrawal of necessary triage.

### 7.4 Implementation and equity

Evaluation can use RE-AIM, the original CFIR domains, and implementation-outcome distinctions to examine reach, adoption, context, fidelity, feasibility, maintenance, and cost (40–42). Denominators should include eligible people who never enroll. Prespecified analyses should compare effects and harms by language, literacy, disability, deprivation, and service access, while avoiding underpowered claims of equivalence.

P5 would be weakened if reliably measured differences in opportunity do not modify the expected skill-to-action relationship with sufficient precision. A separate equity claim would fail if a program’s average benefit accompanies a clinically meaningful widening of disparities in timely care, functioning, or monitoring burden. A protocol should define acceptable between-group differences before analysis. Lack of statistically significant interaction is not evidence of equal benefit. Access failures call for service redesign and retesting, rather than an assumption that individuals need stronger motivation.

## 8 Clinical and public-health application

### 8.1 A brief consultation workflow

The proposed six-step sequence is: screen for danger; reconstruct the relevant timeline; compare sources of evidence; agree one feasible action; specify feedback and escalation; and review without blame. It is a workflow proposal, not a validated five-minute instrument. In a 5–10-min consultation, a short pre-visit form can record the main functional change, current interpretation, previous action, and access barrier. The clinician uses the encounter for safety assessment, one decision, and a documented follow-up plan. Urgent or complex presentations require the appropriate longer assessment or referral.

A low-risk educational action plan might be: complete a one-minute record of one agreed symptom and its effect on an ordinary activity after the evening meal for seven days, then review it with the designated professional. An accessible alternative is a paper tick-box or a scheduled telephone check if writing or smartphone use is difficult. Stop the optional recording and seek the specified assessment if the safety criteria occur; reduce or stop it if recording increases distress. This illustrative amount and schedule are study-design choices, not a treatment prescription or a universal monitoring recommendation.

Education should assess whether people can describe a change, distinguish evidence from interpretation, choose an achievable action, and recognize when professional help is needed. Teach-back and accessible language can identify misunderstanding. Success is calibrated action and equitable access, not maximum body attention or adherence to advice that cannot realistically be followed.

### 8.2 Cultural adaptation and shared responsibility

An individual’s account may involve family responsibilities, traditional medicine, collective decision-making, or meanings of the body that differ from biomedical categories. Adaptation should begin with interviews and cognitive testing of measures, involving patients, families when invited, community representatives, and clinicians. Translated wording alone does not establish measurement equivalence. Researchers should identify who notices change, who interprets it, who controls resources, and who can authorize or facilitate care, without presuming that these roles belong to one autonomous individual.

Traditional or complementary practices should be discussed respectfully and recorded as potential exposures or actions, including relevant interactions and delays. Cultural meaning need not be dismissed to maintain evidence-based safety boundaries. Shared plans should preserve the person’s preferences and privacy while specifying professional accountability. Where services are scarce, a referral recommendation requires an available route, transport or communication support, and a responsible follow-up contact. Lower access does not justify relaxing the definition of clinically indicated care. WHO’s self-care approach includes individuals, families, communities, and links to health-system support (43). ISRL must be tested as an adaptation to these relationships rather than assumed to be culturally universal.

## 9 Boundaries and limitations

ISRL does not propose one cause of all disease. Stress, beliefs, and attention may influence experience and behavior but should not be used to blame people for cancer, autoimmune disease, or other pathology. Evidence on stress and cancer does not justify attributing an individual’s cancer to their thoughts (44). Biomedical investigation and indicated treatment retain priority.

The present synthesis is selective, several mechanisms overlap, and some supporting evidence is observational or indirect. The source audit distinguishes bibliographic verification from abstract-level and full-text support; a correct DOI alone cannot establish a claim. No proposed measurement threshold, workflow, or intervention has been validated as an ISRL instrument. Restricting the initial population, decision, and time window limits flexibility to accommodate any result, but also limits generalization. Cross-disciplinary integration is a research aspiration rather than proof that a broad model performs better (45).

Repeated monitoring may heighten vigilance, distress, cost, or exclusion. The WHO emphasis on enabling conditions and support for self-care (43) is used here to motivate a burden-sensitive design; that guidance is not itself evidence that this particular monitoring schedule causes anxiety. The hypothesis must be tested through distress measures, burden, withdrawal, and participant reports. Privacy, the option to pause, and access to ordinary care should not depend on completing monitoring. Repeated failure of the core comparative predictions requires simplification or abandonment within the tested setting, not indefinite addition of moderators.

## 10 Discussion

ISRL organizes a clinically relevant question: how does bodily information become an action that is appropriate to risk and feasible in context? Its potential value lies in the explicit measurement of transitions and failure points. A new name, a six-part diagram, or a safety banner is insufficient evidence of added value. The strongest alternative already combines illness representations, capability, opportunity, and triage; any claim of improvement must survive that comparison.

Operational safety makes the proposal more accountable but also more demanding. Services must specify who decides, which guideline applies, how referrals are completed, and how missed and unnecessary escalation are counted. Likewise, construct separation requires more than differently named questionnaires. Independent tasks, adjudication, uncertainty estimates, and out-of-sample prediction are prerequisites to claims about flexibility or proportionality. Cultural and material constraints are part of these tests, not optional adjustments after favorable average results.

The proposed research sequence proceeds from measurement and observational safety assessment to bounded temporal prediction, comparative intervention, and implementation. Failure at an earlier stage should change or stop the later program. Evidence supporting a useful intervention may still fail to support a distinct ISRL theory, and evidence supporting a prediction may still be insufficient for deployment. This separation protects both scientific interpretation and patients.

## 11 Conclusion

The ISRL is a provisional account of transitions from bodily signals to feasible, proportionate action. It links functional, subjective, contextual, and clinical information with condition-specific safety and professional responsibility. Its justification depends on measurable constructs and worthwhile improvements over a simpler active comparator, without added delay, unnecessary escalation, distress, or inequity. The framework should be revised or rejected within settings where those tests fail.

## Data Availability

The original contributions presented in the study are included in the article/Supplementary material, further inquiries can be directed to the corresponding author/s.
