# Supplementary material for: From bodily signals to safe action: an interoceptive self-regulation loop for whole-person health
Source: Front Public Health. 2026 Oct 15;14:1981905. doi: 10.3389/fpubh.2026.1981905 (PMC13720441; doi:10.3389/fpubh.2026.1981905)
Supplement: Supplementary file 1 [file Data_Sheet_1.DOCX]

Supplementary Material S1

# Search transparency and citation verification

This supplement accompanies the revision of manuscript 1981905. It documents revision-stage search activity, the conceptual synthesis, and the evidence inspected for each retained or added reference. All 41 references from the submitted manuscript have identifiable bibliographic or official records. Three guidance sources and one psychometric study were added. The existence of a record, its metadata, an abstract-level claim check, and inspection of relevant full-text passages are distinct levels of verification.

# 1 Scope of verification

Initial checks were performed on 28 September 2026, followed by focused full-text checks on 29 September 2026, using Crossref publisher metadata, PubMed/Europe PMC records, selected accessible full-text passages, and official WHO, NCI and NICE pages. Relevant abstracts were used where full text was not inspected. These checks do not constitute full-text verification of every reference, independent replication, or a systematic assessment of risk of bias. Broad conceptual claims were limited to what the inspected evidence supports; sources were not used as empirical validation of ISRL. The record should be read together with the complete revised bibliography.

The main corrections concern original reference 9 (Simmons JM), original reference 25 (the NCI title and displayed review year), and original reference 6 (an undated WHO topic page). The complete DOI for original reference 21 is retained. The 2018 PREDIMED report is explicitly described as the republished analysis with protocol deviations. Updated discussion of WPHI includes an additional 2026 psychometric study.

The follow-up inspected relevant full-text sections for 11 additional sources. The current record distinguishes 18 sources checked against relevant full-text passages, 10 official guidance pages, and 17 sources checked through abstracts or introductory excerpts and bibliographic records. These categories describe the material inspected, not equivalent levels of evidence. Original references 16 and 30 remain priority full-text gaps; their retained claims are restricted to the published abstracts. The separately logged PubMed searches below remain dated 28 September 2026.

# 2 Revision-stage PubMed searches

These searches were executed for the revision and are not claimed to reconstruct the exact historical strings of the initial narrative mapping. The publication-date window was 1 January 1900 through 28 September 2026; no language filter was applied in the queries. PubMed ESearch used retmax=20 and sort=relevance. The returned total is not a screened, included, or deduplicated study count. Only the first 20 identifiers per query, or all identifiers if fewer, were retrieved. Titles were inspected for conceptual relevance and selected abstracts were examined; no independent duplicate screening or systematic exclusion ledger was conducted. One directly relevant additional WPHI psychometric study was incorporated. Unselected search results must not be interpreted as evidence against other approaches.

## Search 1

(interoception[Title/Abstract]) AND (self-regulation[Title/Abstract] OR appraisal[Title/Abstract] OR "health behavior"[Title/Abstract]) AND ("1900/01/01"[Date - Publication] : "2026/09/28"[Date - Publication])

Execution date: 2026-09-28. Returned records: 168. Identifiers retrieved: 20.

Retrieved PMIDs: 34267621, 25783221, 27522011, 8178539, 29748924, 41231765, 27445681, 42306847, 41825581, 41479973, 41223132, 40912313, 40898753, 40800948, 39505563, 39419923, 39032583, 38663458, 37447320, 36696380.

## Search 2

("whole person health"[Title/Abstract] OR "whole-person health"[Title/Abstract]) AND (measurement[Title/Abstract] OR index[Title/Abstract] OR interoception[Title/Abstract]) AND ("1900/01/01"[Date - Publication] : "2026/09/28"[Date - Publication])

Execution date: 2026-09-28. Returned records: 18. Identifiers retrieved: 18.

Retrieved PMIDs: 42630376, 41231765, 42634694, 41099610, 40756628, 39525287, 42253878, 42221534, 40180567, 40001193, 39791185, 34069544, 42221536, 40907847, 39514490, 42310850, 30933607, 18668176.

## Search 3

(self-management[Title/Abstract]) AND ("safety netting"[Title/Abstract] OR "red flags"[Title/Abstract] OR triage[Title/Abstract]) AND ("1900/01/01"[Date - Publication] : "2026/09/28"[Date - Publication])

Execution date: 2026-09-28. Returned records: 110. Identifiers retrieved: 20.

Retrieved PMIDs: 32643252, 35379241, 42612039, 42561632, 41289584, 39819907, 39652845, 39284181, 38917452, 38805348, 37952276, 37851498, 37436003, 37372694, 37235721, 36293885, 36238753, 36114586, 35777943, 35506491.

The source audit additionally used DOI-based and exact-source lookups and official guidance pages; those lookups are not counted as results of the three PubMed queries. This selective synthesis does not claim exhaustive literature coverage or a reproducible count of all sources screened in the initial manuscript preparation.

# 3 Conceptual synthesis

Supplementary Figure S1. Source frameworks inform the separation of states, interpretations, actions, and context. ISRL then makes comparative predictions; the integration itself is not evidence of validity. The named frameworks are described in main-text Section 3 and Table 1.

# 4 Source-by-source verification

Original numbers refer to the submitted PDF. Revised numbers follow first appearance in the revised manuscript. Added entries have no original number. Each record states the inspected evidence and the bounded use of that evidence; metadata and claim support should be evaluated separately.

## Original 1 → revised 4

World Health Organization. Healthy diet. (2026). Available at: https://www.who.int/news-room/fact-sheets/detail/healthy-diet (accessed 28 September 2026).

Evidence inspected: Official guidance/page.

Official guidance supports dietary-pattern context, not dietary treatment of an unexplained symptom. Page dated 26 January 2026; revision access date recorded.

## Original 2 → revised 5

World Health Organization. Physical activity. (2024). Available at: https://www.who.int/news-room/fact-sheets/detail/physical-activity (accessed 28 September 2026).

Evidence inspected: Official guidance/page.

Official guidance supports physical activity as a health-related behavior. No specific ISRL dose or outcome is inferred. Page dated 26 June 2024.

## Original 3 → revised 6

Lloyd-Jones DM, Allen NB, Anderson CAM, Black T, Brewer LC, Foraker RE, et al. Life’s Essential 8: Updating and Enhancing the American Heart Association’s Construct of Cardiovascular Health: A Presidential Advisory From the American Heart Association. Circulation. (2022) 146:e18–e43. doi: 10.1161/CIR.0000000000001078

Evidence inspected: Abstract and bibliographic record.

Publisher abstract describes diet, activity, sleep and clinical metrics, with social and psychological context. Full article not inspected; reference title standardized from the publisher record.

Source link: https://doi.org/10.1161/CIR.0000000000001078

## Original 4 → revised 7

Watson NF, Badr MS, Belenky G, Bliwise DL, Buxton OM, Buysse D, et al. Recommended Amount of Sleep for a Healthy Adult: A Joint Consensus Statement of the American Academy of Sleep Medicine and Sleep Research Society. Sleep. (2015) 38:843-844. doi: 10.5665/sleep.4716

Evidence inspected: Abstract and bibliographic record.

Consensus record supports adequate adult sleep as a health domain. It does not support a universal bedtime or an ISRL-specific causal effect.

Source link: https://doi.org/10.5665/sleep.4716

## Original 5 → revised 8

Furman D, Campisi J, Verdin E, Carrera-Bastos P, Targ S, Franceschi C, et al. Chronic inflammation in the etiology of disease across the life span. Nat Med. (2019) 25:1822-1832. doi: 10.1038/s41591-019-0675-0

Evidence inspected: Abstract and bibliographic record.

Perspective abstract supports links between social, environmental and lifestyle factors and chronic inflammation. It does not establish one common cause of all disease.

Source link: https://doi.org/10.1038/s41591-019-0675-0

## Original 6 → revised 9

World Health Organization. Social determinants of health. (n.d.). Available at: https://www.who.int/health-topics/social-determinants-of-health (accessed 28 September 2026).

Evidence inspected: Official guidance/page.

Official WHO topic page supports social determinants as contextual influences. A unique publication date is not displayed; replaced the unsupported 2025 label with n.d. and a current access date.

## Original 7 → revised 10

Craig AD. How do you feel? Interoception: the sense of the physiological condition of the body. Nat Rev Neurosci. (2002) 3:655-666. doi: 10.1038/nrn894

Evidence inspected: Abstract and bibliographic record.

Review abstract supports the account of internal bodily-state representation. It is foundational theory, not evidence validating this decision workflow.

Source link: https://doi.org/10.1038/nrn894

## Original 8 → revised 11

Khalsa SS, Adolphs R, Cameron OG, Critchley HD, Davenport PW, Feinstein JS, et al. Interoception and Mental Health: A Roadmap. Biol Psychiatry Cogn Neurosci Neuroimaging. (2018) 3:501-513. doi: 10.1016/j.bpsc.2017.12.004

Evidence inspected: Relevant full-text passages and bibliographic record.

Follow-up on 29 September 2026: conceptual sections and Tables 2–3 distinguish attention, detection, discrimination, accuracy, insight, sensibility and self-report, with only partial overlap among measures. Supports separate constructs; does not validate the proposed ISRL task or clinical decisions.

Source link: https://doi.org/10.1016/j.bpsc.2017.12.004

## Original 9 → revised 12

Chen WG, Schloesser D, Arensdorf AM, Simmons JM, Cui C, Valentino R, et al. The Emerging Science of Interoception: Sensing, Integrating, Interpreting, and Regulating Signals within the Self. Trends Neurosci. (2021) 44:3-16. doi: 10.1016/j.tins.2020.10.007

Evidence inspected: Relevant full-text passages and bibliographic record.

Follow-up on 29 September 2026: definition, unified framework and function sections support sensing, interpretation, integration and regulation of internal signals. The paper includes conscious and nonconscious processes. Simmons JM is confirmed; no direct ISRL efficacy or measurement validation is inferred.

Source link: https://doi.org/10.1016/j.tins.2020.10.007

## Original 10 → revised 13

Chen WG, Langevin HM. Interoception as a central mechanism in Whole Person Health. PLoS Biol. (2025) 23:e3003487. doi: 10.1371/journal.pbio.3003487

Evidence inspected: Relevant full-text passages and bibliographic record.

Relevant full-text passages describe whole-person health and the 4M research program. ISRL is compared with this existing contribution; it does not claim to discover that interoception matters.

Source link: https://doi.org/10.1371/journal.pbio.3003487

## Original 11 → revised 14

Sterling P. Allostasis: a model of predictive regulation. Physiol Behav. (2012) 106:5-15. doi: 10.1016/j.physbeh.2011.06.004

Evidence inspected: Abstract and bibliographic record.

Abstract supports anticipatory regulation and allostasis. No numerical clinical threshold is taken from this conceptual article.

Source link: https://doi.org/10.1016/j.physbeh.2011.06.004

## Original 12 → revised 15

McEwen BS. Protective and damaging effects of stress mediators. N Engl J Med. (1998) 338:171-179. doi: 10.1056/NEJM199801153380307

Evidence inspected: Publisher abstract/introductory excerpt and bibliographic record.

Publisher introductory excerpt and bibliographic record concern protective and damaging stress responses and allostatic load. Full text not inspected; used only for the broad conceptual background.

Source link: https://doi.org/10.1056/NEJM199801153380307

## Original 13 → revised 16

Guidi J, Lucente M, Sonino N, Fava GA. Allostatic Load and Its Impact on Health: A Systematic Review. Psychother Psychosom. (2021) 90:11-27. doi: 10.1159/000510696

Evidence inspected: Abstract and bibliographic record.

Systematic-review abstract supports the relationship of allostatic load to clinical and population outcomes. It is not intervention evidence for ISRL.

Source link: https://doi.org/10.1159/000510696

## Original 14 → revised 17

Parker HW, Abreu AM, Sullivan MC, Vadiveloo MK. Allostatic Load and Mortality: A Systematic Review and Meta-Analysis. Am J Prev Med. (2022) 63:131-140. doi: 10.1016/j.amepre.2022.02.003

Evidence inspected: Abstract and bibliographic record.

Meta-analysis abstract supports an association between higher allostatic load and mortality, with heterogeneity. Revision retains associative language.

Source link: https://doi.org/10.1016/j.amepre.2022.02.003

## Original 15 → revised 18

Mehling WE, Acree M, Stewart A, Silas J, Jones A. The Multidimensional Assessment of Interoceptive Awareness, Version 2 (MAIA-2). PLoS One. (2018) 13:e0208034. doi: 10.1371/journal.pone.0208034

Evidence inspected: Relevant full-text passages and bibliographic record.

Follow-up on 29 September 2026: Methods, Results and Limitations describe the 37-item, eight-scale MAIA-2 and its self-report limitations. Supports a multidimensional questionnaire; does not establish objective interoceptive accuracy, clinical diagnosis, or validity of the new flexibility/proportionality measures. These boundaries are explicit in Section 3.1.

Source link: https://doi.org/10.1371/journal.pone.0208034

## Original 16 → revised 19

Leventhal H, Phillips LA, Burns E. The Common-Sense Model of Self-Regulation (CSM): a dynamic framework for understanding illness self-management. J Behav Med. (2016) 39:935-946. doi: 10.1007/s10865-016-9782-2

Evidence inspected: Abstract and bibliographic record.

Abstract explicitly includes illness representations, action planning and continuous feedback. Revised comparison acknowledges these existing CSM features. Follow-up on 29 September 2026 reached the publisher abstract and reference list, not the full article. The retained CSM description is limited to features explicitly stated in the abstract; full-text confirmation remains outstanding.

Source link: https://doi.org/10.1007/s10865-016-9782-2

## Original 17 → revised 20

Gross JJ. The Emerging Field of Emotion Regulation: An Integrative Review. Review of General Psychology. (1998) 2:271-299. doi: 10.1037/1089-2680.2.3.271

Evidence inspected: Abstract and bibliographic record.

Publisher-supplied abstract in Crossref supports five stages of emotion regulation. Full article not inspected; use limited to the process-model distinction.

Source link: https://doi.org/10.1037/1089-2680.2.3.271

## Original 18 → revised 21

McRae K, Gross JJ. Emotion regulation. Emotion. (2020) 20:1-9. doi: 10.1037/emo0000703

Evidence inspected: Abstract and bibliographic record.

Review abstract supports context-dependent emotion regulation and reappraisal. It does not establish the proposed ISRL updating task's validity.

Source link: https://doi.org/10.1037/emo0000703

## Original 19 → revised 22

Michie S, van Stralen MM, West R. The behaviour change wheel: a new method for characterising and designing behaviour change interventions. Implement Sci. (2011) 6:42. doi: 10.1186/1748-5908-6-42

Evidence inspected: Relevant full-text passages and bibliographic record.

Follow-up on 29 September 2026: the COM-B definitions and Figure 1 describe reciprocal influences and both reflective and automatic motivation. Section 3.2 now retains these motivational processes explicitly; the comparative design does not attribute existing COM-B features to ISRL.

Source link: https://doi.org/10.1186/1748-5908-6-42

## Original 20 → revised 23

Hennessy EA, Johnson BT, Acabchuk RL, McCloskey K, Stewart-James J. Self-regulation mechanisms in health behavior change: a systematic meta-review of meta-analyses, 2006-2017. Health Psychol Rev. (2020) 14:6-42. doi: 10.1080/17437199.2019.1679654

Evidence inspected: Relevant full-text passages and bibliographic record.

Follow-up on 29 September 2026: Methods, mechanism results and Discussion distinguish tests of self-regulation change from comparisons of intervention components. Only four meta-analyses directly tested the mechanism relationship; components were not consistently effective across populations and behaviors. Supports qualified language, not a universal causal mechanism.

Source link: https://doi.org/10.1080/17437199.2019.1679654

## Original 21 → revised 27

Sletten TL, Weaver MD, Foster RG, Gozal D, Klerman EB, Rajaratnam SMW, et al. The importance of sleep regularity: a consensus statement of the National Sleep Foundation sleep timing and variability panel. Sleep Health. (2023) 9:801-820. doi: 10.1016/j.sleh.2023.07.016

Evidence inspected: Abstract and bibliographic record.

Consensus abstract and metadata support sleep-timing regularity. Complete DOI is 10.1016/j.sleh.2023.07.016; that suffix was already visible in the supplied PDF and is preserved.

Source link: https://doi.org/10.1016/j.sleh.2023.07.016

## Original 22 → revised 28

Windred DP, Burns AC, Lane JM, Saxena R, Rutter MK, Cain SW, et al. Sleep regularity is a stronger predictor of mortality risk than sleep duration: A prospective cohort study. Sleep. (2024) 47:zsad253. doi: 10.1093/sleep/zsad253

Evidence inspected: Abstract and bibliographic record.

Cohort results support an association between sleep regularity and mortality. Revision avoids inferring mortality reduction from changing sleep timing.

Source link: https://doi.org/10.1093/sleep/zsad253

## Original 23 → revised 29

Estruch R, Ros E, Salas-Salvadó J, Covas MI, Corella D, Arós F, et al. Primary Prevention of Cardiovascular Disease with a Mediterranean Diet Supplemented with Extra-Virgin Olive Oil or Nuts. N Engl J Med. (2018) 378:e34. doi: 10.1056/NEJMoa1800389

Evidence inspected: Abstract and bibliographic record.

Abstract describes the 2018 republished PREDIMED analysis and original protocol deviations. Revision explicitly retains those qualifications and the defined high-risk population.

Source link: https://doi.org/10.1056/NEJMoa1800389

## Original 24 → revised 30

World Health Organization. WHO guidelines on physical activity and sedentary behaviour. (2020). Available at: https://www.who.int/publications/i/item/9789240015128 (accessed 28 September 2026).

Evidence inspected: Official guidance/page.

Official WHO guideline page identifies the 2020 guidance and target populations. Used for the broad role and population dependence of activity, not for a newly prescribed dose.

## Original 25 → revised 44

National Cancer Institute. Stress and Cancer. (2022). Available at: https://www.cancer.gov/about-cancer/coping/feelings/stress-fact-sheet (accessed 28 September 2026).

Evidence inspected: Official guidance/page.

Official page is titled Stress and Cancer and displays Reviewed 21 October 2022. Corrected submitted title/year; retained only for the limited anti-blame boundary, with disease-specific digressions removed.

## Original 26 → revised 3

World Health Organization. World report on social determinants of health equity. (2025). Available at: https://www.who.int/publications/i/item/9789240107588 (accessed 28 September 2026).

Evidence inspected: Official guidance/page.

Official report overview supports social and structural determinants and equity. Published 6 May 2025; no invented numerical estimate is used.

## Original 27 → revised 32

Brown TM, Brainard GC, Cajochen C, Czeisler CA, Hanifin JP, Lockley SW, et al. Recommendations for daytime, evening, and nighttime indoor light exposure to best support physiology, sleep, and wakefulness in healthy adults. PLoS Biol. (2022) 20:e3001571. doi: 10.1371/journal.pbio.3001571

Evidence inspected: Abstract and bibliographic record.

Consensus abstract support light exposure as a circadian input. Does not establish that changing light resolves unexplained clinical symptoms.

Source link: https://doi.org/10.1371/journal.pbio.3001571

## Original 28 → revised 33

Goyal M, Singh S, Sibinga EMS, Gould NF, Rowland-Seymour A, Sharma R, et al. Meditation programs for psychological stress and well-being: a systematic review and meta-analysis. JAMA Intern Med. (2014) 174:357-368. doi: 10.1001/jamainternmed.2013.13018

Evidence inspected: Relevant full-text passages and bibliographic record.

Follow-up on 29 September 2026: efficacy results, Harms and Discussion support small improvements in selected symptoms versus nonspecific active controls, not superiority over other active treatments. Harms were reported in only a small subset of trials. Section 5.6 now specifies comparator and limited harms evidence. Proposed ISRL safeguards are not represented as tested by this review.

Source link: https://doi.org/10.1001/jamainternmed.2013.13018

## Original 29 → revised 35

Pitcher MH, Langevin HM. The Whole Person Health Index: A Self-Reported Tool to Assess Whole Person Health. Glob Adv Integr Med Health. (2026) 15:27536130261455354. doi: 10.1177/27536130261455354

Evidence inspected: Relevant full-text passages and bibliographic record.

Viewpoint and relevant full-text description introduce the WPHI. The source is not an ISRL validation study. Added a distinct 2026 psychometric paper to avoid implying that no validation evidence exists.

Source link: https://doi.org/10.1177/27536130261455354

## Original 30 → revised 37

Mulder J, Boelens M, van der Velde LA, Brust M, Kiefte-de Jong JC. The role of interoception in lifestyle factors: A systematic review. Neurosci Biobehav Rev. (2025) 169:106018. doi: 10.1016/j.neubiorev.2025.106018

Evidence inspected: Abstract and bibliographic record.

Systematic-review abstract describes limited-quality evidence across lifestyle factors and calls for longitudinal work. Supports uncertainty, not a confirmed causal pathway. Follow-up on 29 September 2026 did not retrieve the published full text. An earlier preprint and conference abstract were not substituted for the cited journal version; the retained claim is limited to the published abstract.

Source link: https://doi.org/10.1016/j.neubiorev.2025.106018

## Original 31 → revised 43

World Health Organization. Self-care for health and well-being. (2026). Available at: https://www.who.int/news-room/fact-sheets/detail/self-care-health-interventions (accessed 28 September 2026).

Evidence inspected: Official guidance/page.

Official WHO page supports individual, family and community self-care with health-system support. It is not cited as direct evidence that the proposed ISRL monitoring schedule induces anxiety.

## Original 32 → revised 24

Barrett LF, Simmons WK. Interoceptive predictions in the brain. Nat Rev Neurosci. (2015) 16:419-429. doi: 10.1038/nrn3950

Evidence inspected: Relevant full-text passages and bibliographic record.

Follow-up on 29 September 2026: active-inference discussion and EPIC hypotheses connect prediction error, attention and the expected reliability of sensory signals. This is a theoretical account, not validation of a confidence questionnaire. Section 3.2 now identifies confidence ratings as candidate behavioral indicators whose relationship to computational precision requires testing.

Source link: https://doi.org/10.1038/nrn3950

## Original 33 → revised 25

Seth AK, Friston KJ. Active interoceptive inference and the emotional brain. Philos Trans R Soc Lond B Biol Sci. (2016) 371:20160007. doi: 10.1098/rstb.2016.0007

Evidence inspected: Relevant full-text passages and bibliographic record.

Follow-up on 29 September 2026: prediction-error and precision sections distinguish computational reliability (inverse variance) from its proposed neural implementation. They do not validate the ISRL vignette score. The manuscript retains this distinction and avoids equating self-reported confidence with neural precision.

Source link: https://doi.org/10.1098/rstb.2016.0007

## Original 34 → revised 26

Pezzulo G, Rigoli F, Friston K. Active Inference, homeostatic regulation and adaptive behavioural control. Prog Neurobiol. (2015) 134:17-35. doi: 10.1016/j.pneurobio.2015.09.001

Evidence inspected: Relevant full-text passages and bibliographic record.

Follow-up on 29 September 2026: sections on action, allostasis and hierarchical control connect autonomic, behavioral and goal-directed regulation over different timescales. Supports theoretical background, not a validated clinical workflow or proof of the proposed decision measures.

Source link: https://doi.org/10.1016/j.pneurobio.2015.09.001

## Original 35 → revised 38

DiGuiseppi G, Rodriguez A, Qureshi N, Zeng C, Coulter ID, Hays RD, et al. Measuring Whole Person Health: A Scoping Review. J Integr Complement Med. (2025) 31:684-704. doi: 10.1089/jicm.2024.0817

Evidence inspected: Relevant full-text passages and bibliographic record.

Follow-up on 29 September 2026: Results, Table 3 and Discussion describe six self-report measures and less environmental/socioeconomic coverage relative to conceptual models in that review. Limitations include targeted WPH terminology, a restricted publication window and limited evidence on responsiveness. Section 7.2 now states the comparison precisely rather than generalizing it to all health measures.

Source link: https://doi.org/10.1089/jicm.2024.0817

## Original 36 → revised 39

Alvarez-Ambrosio M, Chausa P, Moreno-Blanco D, Roca-Ventura A, Oropesa I, Cattaneo G, et al. Digital phenotyping for assessment and prediction of interoception, chronic stress, and self-regulation in adults: a scoping review. Front Digit Health. (2026) 8:1710891. doi: 10.3389/fdgth.2026.1710891

Evidence inspected: Relevant full-text passages and bibliographic record.

Abstract and relevant results passages describe 18 included studies, of which two assessed interoception, and heterogeneous analytical performance. Revision avoids treating wearable classifications as clinical diagnoses.

Source link: https://doi.org/10.3389/fdgth.2026.1710891

## Original 37 → revised 31

Aitken A, Sawyer A, Iwasaki A, Krumholz HM, Preston R, Calcraft P, et al. Digital physiological biomarkers predict within-person symptom changes in complex chronic illness. NPJ Digit Med. (2026) 9:257. doi: 10.1038/s41746-026-02543-3

Evidence inspected: Relevant full-text passages and bibliographic record.

Abstract and relevant methods/results describe within-person biometrics and symptom prediction in complex chronic illness. Supports a limited feasibility example and cognitive-fatigue outcome context, not general ISRL causality or clinical validation.

Source link: https://doi.org/10.1038/s41746-026-02543-3

## Original 38 → revised 40

Glasgow RE, Vogt TM, Boles SM. Evaluating the public health impact of health promotion interventions: the RE-AIM framework. Am J Public Health. (1999) 89:1322-1327. doi: 10.2105/AJPH.89.9.1322

Evidence inspected: Abstract and bibliographic record.

Framework abstract supports reach, efficacy, adoption, implementation and maintenance as evaluation domains. It does not validate this proposed implementation.

Source link: https://doi.org/10.2105/AJPH.89.9.1322

## Original 39 → revised 41

Damschroder LJ, Aron DC, Keith RE, Kirsh SR, Alexander JA, Lowery JC. Fostering implementation of health services research findings into practice: a consolidated framework for advancing implementation science. Implement Sci. (2009) 4:50. doi: 10.1186/1748-5908-4-50

Evidence inspected: Relevant full-text passages and bibliographic record.

Original 2009 framework and relevant conceptual passages support the original CFIR domains. Revision labels them as original rather than claiming that the 2009 terminology is the latest version.

Source link: https://doi.org/10.1186/1748-5908-4-50

## Original 40 → revised 42

Proctor E, Silmere H, Raghavan R, Hovmand P, Aarons G, Bunger A, et al. Outcomes for implementation research: conceptual distinctions, measurement challenges, and research agenda. Adm Policy Ment Health. (2011) 38:65-76. doi: 10.1007/s10488-010-0319-7

Evidence inspected: Relevant full-text passages and bibliographic record.

Abstract and relevant conceptual passages distinguish implementation outcomes from clinical and service outcomes. Supports feasibility, acceptability, fidelity and cost as separate outcomes.

Source link: https://doi.org/10.1007/s10488-010-0319-7

## Original 41 → revised 45

Buvat I, Aarntzen E, Badawi RD, Ballesta A, Bock C, Chuah CN, et al. Cross-disciplinary methodologies for whole-person research - insights from EMPOWER2024. Npj Imaging. (2026) 4:19. doi: 10.1038/s44303-026-00151-0

Evidence inspected: Relevant full-text passages and bibliographic record.

Meeting-report abstract and relevant full-text discussion support cross-disciplinary methodology as a research agenda. It is not an empirical demonstration of a superior ISRL model.

Source link: https://doi.org/10.1038/s44303-026-00151-0

## Added source → revised 34

National Institute for Health and Care Excellence. Recent-onset chest pain of suspected cardiac origin: assessment and diagnosis. Clinical guideline CG95. Updated 30 November 2016. (2016). Available at: https://www.nice.org.uk/guidance/cg95/chapter/recommendations (accessed 28 September 2026).

Evidence inspected: Official guidance/page.

Official NICE CG95 guidance. Relevant recommendations 1.2.1.7–1.2.1.8, 1.2.1.12–1.2.1.13 and 1.2.2.5 were inspected and rechecked on 29 September 2026. The adult example distinguishes emergency from same-day assessment, retains other serious causes, and illustrates when monitoring must stop; it is not a complete or universally transferable protocol.

## Added source → revised 1

World Health Organization. Noncommunicable diseases. (2025). Available at: https://www.who.int/news-room/fact-sheets/detail/noncommunicable-diseases (accessed 28 September 2026).

Evidence inspected: Official guidance/page.

Added WHO noncommunicable-disease fact sheet, dated 25 September 2025. Used for the broad chronic-disease context without introducing an unneeded numerical burden estimate.

## Added source → revised 2

World Health Organization. Health literacy. (2025). Available at: https://www.who.int/news-room/fact-sheets/detail/health-literacy (accessed 28 September 2026).

Evidence inspected: Official guidance/page.

Added WHO health-literacy fact sheet, dated 22 December 2025. Used for the need to make information accessible and actionable; not evidence of ISRL effectiveness.

## Added source → revised 36

Cibelli Hibben K, Ryan VM, Nadreau R, Lamoreaux C, Forrest SE, Scanlon PJ, et al. Assessing the Structural Validity and Measurement Invariance of the Whole Person Health Index. Glob Adv Integr Med Health. (2026) 15:27536130261453376. doi: 10.1177/27536130261453376

Evidence inspected: Relevant full-text passages and bibliographic record.

Follow-up on 29 September 2026: factor-model results, differential item functioning and Discussion support a dominant general factor with mixed strict single-factor fit and age-related differences in the self-rated-health item. Lifestyle and outcome content overlap is discussed by the source; Section 7.2 adds a study-design caution against treating shared predictor/outcome item content as added predictive value. This study does not validate ISRL.

Source link: https://doi.org/10.1177/27536130261453376

# 5 Interpretation of newly proposed tests

The next-day and seven-day windows, example recording schedule, and illustrative construct-correlation criteria in the manuscript are proposed research design choices. They are not validated clinical thresholds or numerical findings from the cited literature. A future protocol must justify these choices, define minimally worthwhile effects and condition-specific harm limits before recruitment, and register its analysis and stopping rules. No human study, pilot validation, or comparative trial is reported in this article.

The refined proposal defines directional discordance patterns, equalizes baseline model flexibility and basic follow-up between trial arms, and separates blinded research adjudication from actual actions and later outcomes. These are safeguards for future study design, not procedures already performed on participants. This refinement did not add a reference or change the scope of the logged searches.
